# The zero responder: a definition and report of current literature

**DOI:** 10.29045/14784726.2024.9.9.2.38

**Published:** 2024-09-01

**Authors:** Eloise Graham, John Hall, Keith Porter

**Affiliations:** Barts Health NHS Trust; Faculty of Pre-Hospital Care, The Royal College of Surgeons of Edinburgh; Faculty of Pre-Hospital Care, The Royal College of Surgeons of Edinburgh

**Keywords:** mass casualty incidents, pre-hospital emergency care, zero responder

## Abstract

The term ‘zero responder’ was initially devised in 2010 to describe those passing by or unharmed in a mass casualty incident, who provide life-saving care for injured persons before qualified professionals arrive. This review aims to determine how the literature defines the role of the zero responder and to explore how they can be better integrated into the emergency response.

Current definitions of the zero responder in a medical setting were found through a literature search of several databases and online libraries using defined search terms. Additionally, a manual search of citations in included articles was performed to yield more results. In total, 16 papers defining the zero responder were included. These definitions were evaluated, and a revised definition was suggested to clarify the role in a medical setting relating to mass casualty incidents.

The role of the zero responder can be facilitated through authority recognition and adequate equipment provision. Familiarisation with the term and role of zero responders among ambulance services is essential for effective collaboration. Further research and clarity on the integration of these two groups is necessary to facilitate effective and safe working between them.

## Introduction

The term ‘first responders’ broadly describes the group of people, generally paramedics or other emergency services, who are first to reach the scene of an incident where there are casualties. However, it is not inclusive of those passing by or unharmed in the incident, who can provide life-saving care for injured persons before the official response arrives; these are the ‘zero responders’.

In the wake of the Manchester Arena Attack, where only one paramedic arrived at the room of the explosion within the first 40 minutes, there is demonstratable need for time-sensitive first aid before organised help arrives (Gardham, 2021). At Manchester, while healthcare professionals with enhanced trauma management and triaging skills were waiting to be mobilised into the venue, members of the public worked alongside unarmed police officers and security staff to treat casualties (Saunders, 2022). In the enquiry, chairman Sir John Saunders described how many of these civilians wished they had been equipped with better first-aid skills (Saunders, 2022). Indeed, one of the 22 victims, John Atkinson, may have survived if basic first aid had been administered sooner (Saunders, 2021a). Mr Atkinson went into hypovolaemic cardiac arrest from catastrophically haemorrhaging leg wounds an hour and 16 minutes after the blast; no medical-grade tourniquet or haemostatic dressings had been applied to his wounds (Saunders, 2021a).

This article aims to explore definitions of the term ‘zero responder’ and determine whether these definitions stand up to modern expectations of the role. Defining the group of people who are unharmed in mass casualty events as zero responders is an important step in ensuring adequate resource planning, first-aid training and equipment provision.

## Scoping review of current literature

A literature search of several databases, carried out on 9 May 2024 and detailed in Table 1, revealed 230 papers (including duplicates) with ‘zero responder’ indexed in subject headings or keywords. Titles were then screened, followed by abstract or full-text screening, and irrelevant papers were excluded. A total of 13 papers, with duplicates excluded, were found from the database search. A manual search of the references of the included articles was then carried out, adding a further three pieces of literature to the evidence base. Overall, 16 papers were included for review.

**Table 1.** The databases searched, search terms used and limitations applied in the literature acquisition.

Database	Search terms	Limits	Results	Relevant results after screening
**Ovid Medline** (1946 to 9 April 2024)	(“zero responder”) ***OR*** (“zero responders”)*** OR*** (zero-responder) ***OR*** (zero-responders) ***OR*** (“zero-order responders”) ***OR*** (“zero-order responders”)	English language	3	0
**Embase** (1974 to 9 April 2024)	(“zero responder”) ***OR*** (“zero responders”)*** OR*** (zero-responder) ***OR*** (zero-responders) ***OR*** (“zero-order responders”) ***OR*** (“zero-order responders”)	English language	3	0
**PsycInfo** (1806 to 9 April 2024)	(“zero responder”) ***OR*** (“zero responders”)*** OR*** (zero-responder) ***OR*** (zero-responders) ***OR*** (“zero-order responders”) ***OR*** (“zero-order responders”)	English language	3	1
**CINAHL Ultimate**	(“zero responder”) ***OR*** (“zero responders”)*** OR*** (zero-responder) ***OR*** (zero-responders) ***OR*** (“zero-order responders”) ***OR*** (“zero-order responders”)	English language	16	0
**Scopus**	(“zero responder”) ***OR*** (“zero responders”)*** OR*** (zero-responder) ***OR*** (zero-responders) ***OR*** (“zero-order responders”) ***OR*** (“zero-order responders”)	English language	41	12
**University of Birmingham library catalogue**	(“zero responder”) ***OR*** (“zero responders”)*** OR*** (zero-responder) ***OR*** (zero-responders) ***OR*** (“zero-order responders”) ***OR*** (“zero-order responders”)	English language	29	4
**Barts Health NHS Trust library catalogue**	(“zero responder”) ***OR*** (“zero responders”)*** OR*** (zero-responder) ***OR*** (zero-responders) ***OR*** (“zero-order responders”) ***OR*** (“zero-order responders”)	English language	135	8
**Total**				**25**
**Total after removing duplicates**				**13**
**Total including manual search results**				**16**

Zero responders appear to have been first described in 2010 by Lemyre et al. (2013). They define zero responders as passers-by of an unfolding emergency, as well as those caught up in the incident but not critically injured themselves, who can ‘act and react’ before official first responders arrive. They emphasise that this group are distinctly unique from first responders, an idea echoed by McAleavy who describes zero responders as culturally different from first responders (McAleavy, 2021). By devising the term, Lemyre et al. aimed to increase zero responder visibility and to argue for their inclusion in emergency planning frameworks to enhance the emergency response. Overall, Lemyre et al. identified the need for a paradigm shift away from the popular belief that members of the public have little means to tangibly enhance the crisis response.

Cocking, another author influential in the field of mass emergency behaviour, expands on Lemyre et al.’s definition of zero responders to describe uninjured bystanders who can treat casualties in mass emergencies (Cocking, 2013; Cocking & Drury, 2014). One of his qualitative papers examines the psychological processes involved when survivors chose to help those injured during the 7/7 London bombings (Cocking, 2013). He found that survivors did not obstruct emergency services but showed group resilience and were keen to assist the emergency response. This idea is further explored by Cocking in his work with Drury on the Hillsborough disaster (Cocking & Drury, 2014). While Hillsborough survivors frequently used the word ‘panic’ to describe the incident, their behaviours matched more with being ‘orderly’ than with any selfish and irrational actions suggesting ‘mass panic’. The paper proposes that this mismatch is due to the entrenchment of the word ‘panic’ in societal discourse and media coverage surrounding unfolding emergencies. The prevalence of disaster myths is further explored by Nogami, who describes zero responders as ‘disaster victims’ who perform ‘life-saving activities’ in ‘postdisaster situations’ (Nogami, 2018). Nogami’s study found that both professional and lay-person groups give statistically significant credit to the misconception that victims panic and are unable to cope after a disaster. Such misconceptions are refuted by experts in crowd behaviour, whose work suggests that zero responders have innovative and adaptive responses to emergencies that facilitate community survival (Au-Yeung et al., 2024; Briones et al., 2019; Drury, 2018; Drury et al., 2009, 2016, 2019; Pearce et al., 2019). Collectively, these papers question the use of the ‘mass panic’ rhetoric as a credible argument against using zero responders as a force willing to save lives.

Cole et al. refer to Lemyre et al.’s definition of zero responders when calling on a surviving crowd to be ‘part of the solution, not the problem’ (Cole et al., 2011). This sentiment is echoed in Duda et al.’s piece, which refers to the zero responder as a means to ‘make up for institutional shortcomings’ in disasters (Duda et al., 2020). It is therefore imperative, Cole et al. argue, to understand which factors affect a zero responder materialising from the crowd, so as to better plan emergency responses for future mass casualty incidents. Similarly, understanding such factors is listed as a research priority in a recent paper by Haghani et al. (2022). One element considered by Cole et al. is the crowd’s awareness of what is *actually* happening during a terrorist incident. Another factor affecting the materialisation of zero responders is their self-assessed competence in delivering life-saving care, as well as the equipment available to them. In a more recent paper by Haghani et al. (2023), zero responders are described as individuals who are present at an emergency but are ‘not trained emergency responders’. However, the authors of this paper would suggest that zero responders may be off-duty medical professionals or civilians with basic first-aid certificates. Indeed, bystanders with first-aid or evacuation training are probably more likely to offer their services than those without.

Nevertheless, Haghani et al. (2023) suggest other ways beyond just medical provision that zero responders can assist in a crowd emergency, including alerting emergency services and helping with evacuation.

## Discussion

### Definition

The term ‘zero responder’ is gaining traction but is yet to be widely popularised. The existing works on zero responders largely quote Lemyre et al.’s definition coined in 2010 (2013). While seminal literature, Lemyre gives no explicit description of what the *role* of the zero responder is, apart from being able to ‘act and react’. Others have expanded on Lemyre’s definition, but no persistently used modern definition has yet been established. Hence, the authors propose the following definition of zero responder to be used henceforth:

The zero-responder is an individual who has witnessed or has been sufficiently uninjured in a multiple casualty incident who is able to provide first aid to those more significantly injured. The zero-responder’s role may also expand beyond medical provision to include initial scene-management, communicating with emergency services, providing immediate emotional support to victims, and more.

### Government/authority recognition

The National Counter Terrorism Security Office (NaCTSO) guidance for the public states that in a terrorist event, they should make themselves safe and alert the authorities. This is encapsulated in their campaign for the public to ‘run, hide, tell’ (National Counter Terrorism Security Office, 2017). However, an analysis of UK emergency planning guidance documents revealed several references to crowd mass panic and vulnerability, with only limited references to the public being active ‘responders’ (Drury et al., 2013). The government and influential authorities must move away from the somewhat paternalistic views that, in an emergency, the public always cause widespread panic and nuisance. Instead, establishing relationships between the zero responder and the official responder before incidents occur could save lives. This is echoed in Lord Kerslake’s report, an independent review of the emergency response to the Manchester Arena Attack, where he recommended that:

All planning assumptions and training in respect of preparing for and responding to terrorist attacks in public places should include realistic contingencies for public involvement in casualty care, treatment and evacuation within all incident zones. (Kerslake, 2018)

While giving evidence to the Manchester Arena inquiry as an expert witness, Brigadier Tim Hodgetts proposed the NaCTSO campaign should be amended to ‘run, hide, tell, and when safe to do so, treat’ (Saunders, 2021b). This idea has been used by Counter Terrorism Policing in collaboration with St John Ambulance to create lesson plans and videos for children aged 11 to 16 for personal, social, health and economic (PSHE) education in schools (St John Ambulance, n.d.). Although this is certainly a step in the right direction, more must be done to promote the importance of treating the injured in mass casualty events, when safe to do so, to the wider population.

### citizenAID

Established in 2016, the charity citizenAID aims to empower the public to help themselves and others if caught up in a multi-casualty incident. citizenAID’s free app offers an accessible step-by-step guide to appropriately triaging causalities, preventing life-threatening haemorrhages and more (citizenAID, n.d.). Additionally, its website offers educational materials and familiarisation videos for both adults and children. The zero responder is a target end user of citizenAID’s app. Ensuring the app is promoted into the public eye through social media and other means would ensure that the public are aware of the initiative in the first instance. This would allow a zero responder to download the app and familiarise themselves with its interface prior to needing to use it.

### PAcT first-aid kit

Analysis of recent major incidents in the UK shows that in the immediate aftermath of such events, a ‘therapeutic vacuum’ prevented casualties receiving rapid medical attention while formal emergency care providers waited for the area to be secured (Park et al., 2020). Public access trauma first-aid kits (PAcT kits) were launched in the summer of 2021 to help minimise the effects of this lag time. These kits, devised by Counter Terrorism Policing, St John’s Ambulance and the Faculty of Pre-Hospital Care, Edinburgh, were derived from existing trauma kits that the City of London police had introduced to public places across the city. Rather than being kits for first responders to use, as these existing trauma kits were, PAcT kits are aimed at zero responders. Each kit contains personal protective equipment, ‘tuff-cut’ scissors, wound dressings of different sizes, tourniquets and a cardiopulmonary resuscitation (CPR) face shield.

PAcT kits have adopted three key principles: being publicly accessible, being user friendly for any person regardless of first aid competence and providing equipment appropriate for treating immediately life-threatening injuries. Training is not required to use PAcT kits, owing to an illustrated aid memoire contained within the kit. To ensure maximal benefit, barriers to PAcT kits being utilised in line with their key principles must be identified and addressed. Such barriers include cost, availability of kits, lack of awareness about the kits among organisations who would benefit from procuring them and exclusion of PAcT kits from Health and Safety Executive regulations. While training is not required to use the kits, basic understanding of first aid would clearly be beneficial. Furthermore, while all public- and private-sector organisations are encouraged to include PAcT kits in their first-aid provisions, it is not mandated.

PAcT kits are a resource that could be utilised by zero responders, but more publicity and awareness must be prioritised for them to have maximal effect in the event of a mass casualty incident.

### Drop bags

Another area of innovation that aims to empower zero responders is drop bags – very small haemorrhage kits that can be ‘dropped’ for civilian use by armed police upon their arrival to the scene (Trewern, 2022). Such kits have been carried by several armed police forces for years but have only recently become mandated for all forces nationally. An example kit might contain one tourniquet, one bandage and one pair of gloves. Unlike PAcT kits, their contents solely address major haemorrhage. Having a kit ‘dropped’ next to a zero responder allows them to immediately start treatment rather than spending time locating first-aid supplies such as PAcT kits. Drop bags may also be used by professional first responders carrying limited kit. However, the potential barriers to successful use of drop bags are similar to those of the PAcT kits; someone with a greater understanding of first aid will probably feel more confident in using them, and likely use them to greater effect.

### Ambulance service

Looking forward, familiarisation of the term and role of zero responders among the ambulance services is key towards effective collaboration. Understanding the distinction and potential grey areas in definition between zero and first responders is part of this. Clearly, the work of paramedics and other first responders with expertise, experience and equipment should not be obstructed by a zero responder. There is also unlikely to be a smooth transition of care with well-structured handovers, but in such extreme circumstances, first responders must be prepared to empower and support zero responders where possible. This might include encouraging volunteers to continue their efforts, supplying further kit where appropriate or offering advice. Ideally, the work of zero responders would free up professional responders to triage or treat the most seriously unwell. Furthermore, paramedics are at risk of moral injury during a mass casualty incident due, in part, to the incredibly difficult decisions they may have to make about who to treat when resources are limited (Gustavsson et al., 2022; Muysewinkel et al., 2024). Increasing the resources available to paramedics by involving zero responders in the disaster response may help lessen any guilt they experience, as patients who would otherwise not have been prioritised in initial triage are at least able to receive some degree of care.

It is, however, important to consider the issue of accountability in the event of deterioration of a patient being cared for by a zero responder. A bystander who has started first aid on a patient assumes a duty of care to the casualty and consequently accepts responsibility for treatment given (Resuscitation Council UK, 2018). Therefore, zero responders must only be providing first aid using skills they feel confident in. Further clarity is required to assure paramedics that advice or equipment given that allows zero responders to act *within* their level of practice would not be held against the paramedic should things go wrong.

Future research exploring the relationship between first and zero responders is essential for understanding how we can facilitate the collaboration of these separate groups. Equally, the views of paramedics on the advantages and potential teething issues of the zero responder role must be acknowledged prior to their widespread endorsement.

### Wider use of the zero responder

This article examines the literature surrounding zero responders in the setting of mass casualty incidents. By defining this specific population, the authors focus on the resources and provisions needed to facilitate the integration of their role into the emergency response for such occurrences. However, the zero responder role has previously been examined and defined in the context of other circumstances, namely, in climate- and weather-related disasters (Cutter, 2018; Glantz & Ramírez, 2018; Ogie & Pradhan, 2019). In such situations, a zero responder is defined in a broader, more holistic sense as someone who not only provides medical assistance, but also repairs broken infrastructure or provides food, water or shelter to other survivors. With the increasing threat of environmental hazards inflicted by climate change on the British population, further research is required to establish how the role of zero responder can be supported in such circumstances (His Majesty’s Government, 2023).

Furthermore, one may consider whether the medical zero responder role can be extended to the population of people who offer first aid in more isolated incidents. Are first aiders who give bystander CPR in cardiac arrests prior to emergency service arrival part of the zero responder cohort? After all, mass casualty incidents are rare, and so recognising and advocating for the role of zero responders in these incidents may be hard to achieve. However, the aforementioned definition has been influenced by evidence on the psychological and behavioural elements identified in mass casualty incidents, and not in more commonplace events. Further research would be required to consider whether this definition of zero responders would stand up to more commonplace use, where the factors affecting whether an individual chooses to provide medical support are likely to be different.

## Conclusion

Through review of published literature, the authors have proposed a modern definition for the term ‘zero responder’. It is hoped that this refined definition will promote its recognition in an official sense to endorse sufficient resource planning, first-aid education and equipment provision. Initiatives such as citizenAID, PAcT first-aid kits and drop bags are excellent resources already in place for the zero responder. Through further innovation and education, zero responders might come to be considered a supplemental emergency service and help to save lives. Further research is needed to understand the behaviours and decisions of zero responders to facilitate integration of their role into the emergency response.

## Author contributions

EG performed the literature search, interpreted the findings and drafted the manuscript. JH and KP devised and supervised the project and reviewed previous manuscript versions. All authors read and approved the final article. EG acts as the guarantor for this article.

## Conflict of interest

KP is a co-founder and trustee of citizenAID.

## Ethics

Not required.

## Funding

None.
